# Characterizing Artificial Intelligence Applications in Cancer Research: A Latent Dirichlet Allocation Analysis

**DOI:** 10.2196/14401

**Published:** 2019-09-15

**Authors:** Bach Xuan Tran, Carl A Latkin, Noha Sharafeldin, Katherina Nguyen, Giang Thu Vu, Wilson W S Tam, Ngai-Man Cheung, Huong Lan Thi Nguyen, Cyrus S H Ho, Roger C M Ho

**Affiliations:** 1 Institute for Preventive Medicine and Public Health Hanoi Medical University Hanoi Vietnam; 2 Bloomberg School of Public Health Johns Hopkins University Baltimore, MD United States; 3 Division of Hematology & Oncology Department of Medicine University of Alabama at Birmingham Birmingham, AL United States; 4 Institute for Cancer Outcomes and Survivorship School of Medicine University of Alabama at Birmingham Birmingham, AL United States; 5 Department of Science, Technology, and Society Stanford University Palo Alto, CA United States; 6 Center of Excellence in Evidence-based Medicine Nguyen Tat Thanh University Ho Chi Minh Vietnam; 7 Alice Lee Centre for Nursing Studies Yong Loo Lin School of Medicine National University of Singapore Singapore Singapore; 8 Center of Excellence in Artiﬁcial Intelligence in Medicine Nguyen Tat Thanh University Ho Chi Minh Vietnam; 9 Information Systems Technology and Design Singapore University of Technology and Design Singapore Singapore; 10 Institute for Global Health Innovations Duy Tan University Da Nang Vietnam; 11 Department of Psychological Medicine National University Hospital Singapore Singapore; 12 Center of Excellence in Behavior Medicine Nguyen Tat Thanh University Ho Chi Minh Vietnam; 13 Institute for Health Innovation and Technology National University of Singapore Singapore Singapore; 14 Department of Psychological Medicine Yong Loo Lin School of Medicine National University of Singapore Singapore Singapore

**Keywords:** scientometrics, cancer, artificial intelligence, global, mapping

## Abstract

**Background:**

Artificial intelligence (AI)–based therapeutics, devices, and systems are vital innovations in cancer control; particularly, they allow for diagnosis, screening, precise estimation of survival, informing therapy selection, and scaling up treatment services in a timely manner.

**Objective:**

The aim of this study was to analyze the global trends, patterns, and development of interdisciplinary landscapes in AI and cancer research.

**Methods:**

An exploratory factor analysis was conducted to identify research domains emerging from abstract contents. The Jaccard similarity index was utilized to identify the most frequently co-occurring terms. Latent Dirichlet Allocation was used for classifying papers into corresponding topics.

**Results:**

From 1991 to 2018, the number of studies examining the application of AI in cancer care has grown to 3555 papers covering therapeutics, capacities, and factors associated with outcomes. Topics with the highest volume of publications include (1) machine learning, (2) comparative effectiveness evaluation of AI-assisted medical therapies, and (3) AI-based prediction. Noticeably, this classification has revealed topics examining the incremental effectiveness of AI applications, the quality of life, and functioning of patients receiving these innovations. The growing research productivity and expansion of multidisciplinary approaches are largely driven by machine learning, artificial neural networks, and AI in various clinical practices.

**Conclusions:**

The research landscapes show that the development of AI in cancer care is focused on not only improving prediction in cancer screening and AI-assisted therapeutics but also on improving other corresponding areas such as precision and personalized medicine and patient-reported outcomes.

## Introduction

### Background

Every year, over 200 million healthy life years are lost because of cancer, making it one of the highest health care burden causing disability and mortality among men and women [1]. Fortunately, many types of cancers can be prevented or effectively treated if patients are diagnosed in a timely manner and offered optimal therapies. In many parts of the world, however, programs for cancer control and prevention are facing multiple barriers because of limited health service infrastructure, availability of treatment options, and health worker capacities.

Artificial intelligence (AI) is considered a disruptive innovation in health and medicine. Over the past six decades, AI has been widely applied to many areas of medical research and clinical practice. The number of published papers on AI and its impacts has been rapidly growing within the research community over the past decade. A bibliometric study has shown that the number of studies on AI applications in medicine has tripled in the past 3 years, with the highest interest in cancer research [2]. Various techniques, such as robotics, machine learning, and artificial neural networks, have been applied to the study of cancer, showing promising improvements in clinical prediction, treatment, and diagnosis. For instance, machine learning techniques in the application of proteomics and genomics could increase precision in estimating survival and inform the selection of therapies [3]. In large populations, the development and application of AI also holds potential in screening for cancer and scaling up treatment services in a timely manner.

### Literature Review

Many approaches and products have been developed to support cancer treatment and for prevention at health facilities and within communities. However, the synthesis of resulting evidence from these efforts is necessary to inform decision making. Some authors have conducted systematic reviews of the performance and effectiveness of AI techniques and products in specific cancers [3-10]. Overall, these reviews found that almost all AI-assisted interventions led to greater effectiveness than conventional approaches. However, insights from these efforts have raised some important points for further exploration. Lisboa et al reviewed predictive models using artificial neural networks and suggested the need for rigorous evaluation of results [4]. In addition, Spelt et al emphasized the importance of justifying the complex structure of datasets and individual factors in these models [5]. Ray et al reviewed the wearable systems for cancer detection and found that cloud computing and long-range communication paradigms are still lacking, and that AI and machine learning should be applied to current products [8]. Other authors affirmed the greater performance of image-based AI applications to breast cancer diagnosis, but few studies have been supported by a high level of evidence. Conducting further clinical research and health technology assessment is recommended.

### Objectives

With the rapid development of technologies, AI-based therapeutics, devices, and systems will be vital innovations in cancer control. To accelerate research and development, it is critical to understand current approaches in the applications of AI in cancer care, multiple disciplines involved, and the trends and establishment of the research landscapes. To our knowledge, none of the previous studies have systematically quantified the development of AI in the bibliographic literature of cancer studies. This study analyzes the global trends, patterns, and development of interdisciplinary landscapes in AI and cancer studies.

## Methods

### Search Strategy

We searched and retrieved all papers related to AI in cancer care on the Web of Science (WOS) that is a Web-based database covering the largest proportion of peer-reviewed literature in this field. The full search strategy has been presented elsewhere [2]. In short, we used a set of predefined search terms related to *artificial intelligence* and *health and medicine* to search the WOS for publications (inclusion step) and then excluded those that did not satisfy our eligibility criteria of publication year from 1991 to 2018 and publications other than articles and reviews (exclusion step). In this analysis, we selected all the documents of retrieved data on AI applications related to *cancer care*.

### Data Extraction

**Figure 1:**
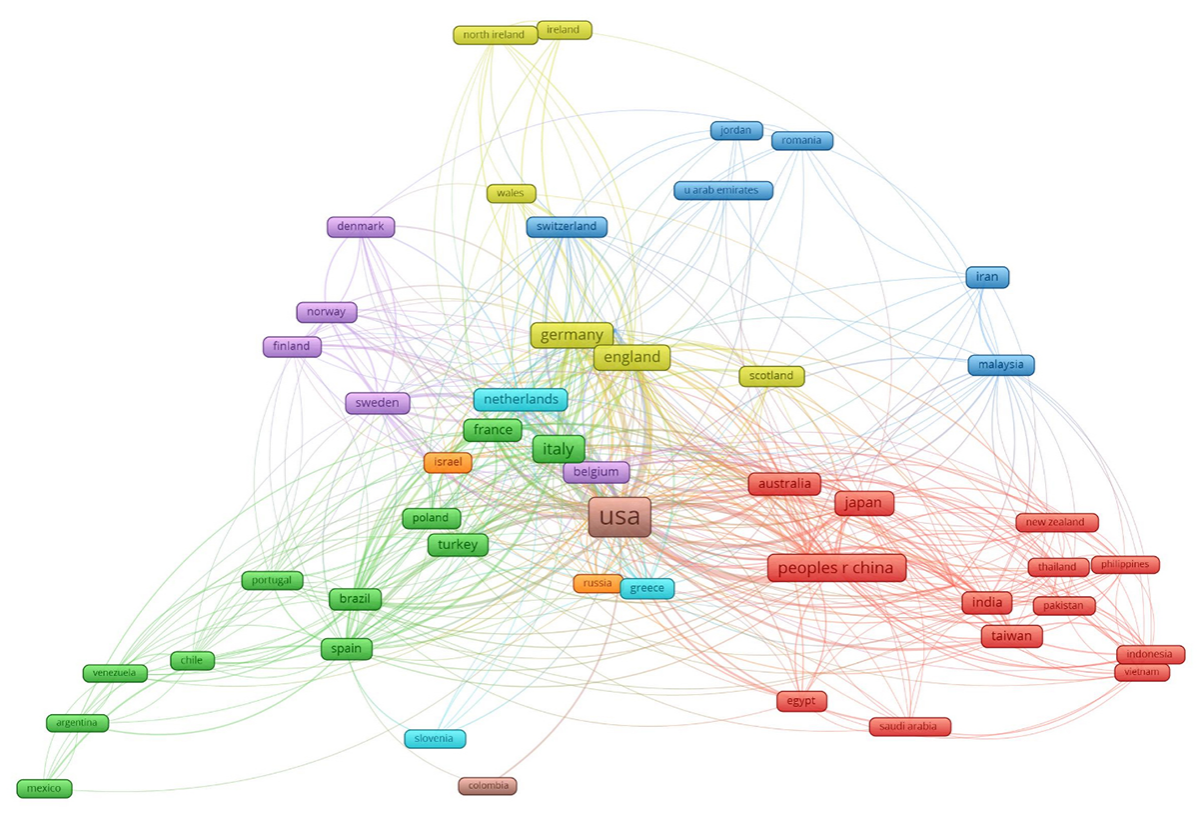
The global networking of 53 countries having at least five coauthorships classified in 8 clusters.

We downloaded all data from the WOS database in .txt format, including all information such as author names, paper title, journals, keywords, affiliations of institutions, the prevalence of citation, categories, and abstracts. All of these data were converted to an Excel file (Microsoft Excel, Microsoft Corporation) for checking the data error. A process of standardization was carried out by 2 researchers to bring together the different names of an author. Then, we filtered all downloaded data using the following criteria: (1) not original articles and reviews, (2) not about cancer and AI, and (3) not in English. Any conflict was solved by discussion (Figure 1). The combined dataset was transferred into Stata (version 14.0, STATA Corporation) for further analysis.

### Data Analysis

Data were resolved based on basic indicators of publication (number of authors, publication years, and main categories), keywords (most common keywords and co-occurrence keywords), citations, usages, and abstracts. After downloading and extracting the data, we applied the descriptive statistical analysis using Stata to calculate country citations and intercountry collaboration. A network graph illustrating the network of countries by sharing the co-authorships was created, along with the author keyword co-occurrence network and countries network. VOSviewer (version 1.6.8, Center for Science and Technology, Leiden University) was used to establish a co-occurrence network and a countries network. The principles of underlying algorithms used by the software for clustering have been documented elsewhere [11-14] For content analysis of the abstracts, we applied the exploratory factor analysis to identify research domains emerging from all content of the abstracts, loadings of 0.4 [15]. The Jaccard similarity index was utilized to identify research topics or terms most frequently co-occurring with each other [16]. Latent Dirichlet Allocation (LDA) was used for classifying papers into corresponding topics [17-21]. The summary of analytical techniques for each data type is presented in Table 1.

**Table 1:** Summary of data analytical techniques.

Type of data	Unit of analysis	Analytical methods	Presentations of results
Authors, keywords, countries	Words	Frequency of co-occurrence	Map of authors keywords clusters
Abstracts	Words	Exploratory factors analyses	Top 50 constructed research domains; clustering map of the landscapes constructed by these domains
Abstracts	Papers	Latent Dirichlet Allocation	10 classifications of research topics
WOS^a^ classification of research areas	WOS research areas	Frequency of co-occurrence	Dendrogram of research disciplines (WOS classification)

^a^WOS: Web of Science.

## Results

### The Number of Published Items and Publication Trend

There has been a rapid increase in the number of studies applying AI to cancer research from 1991 to 2018. In particular, the research productivity of the past 10 years has accounted for over 90.66% (3223/3555) of the total papers. Rates of citation and usage are also growing fast. The mean usage (downloads) in the past 6 months of papers published in the past 1 to 2 years was twice that of those published in the past 3 to 4 years (Table 2).

In Table 3, we examine the study settings mentioned in the abstracts of publications. The bibliography included country settings 749 times, and in those, the United States was mentioned 46.5% of the times. Over 90% of the total settings were in developed countries. Noticeably, 2 countries with large populations, China and India, accounted for 3.3% and 4.4%, respectively.

**Table 2:** General characteristics of publications.

Year published	Total number of papers	Total citations	Mean citation rate per year^a^	Total usage in the last 6 months^b^	Total usage in the last 5 years^b^	Mean use rate for the last 6 months^c^	Mean use rate for the last 5 years^d^
2018	661	809	1.22	2489	3858	3.77	1.17
2017	503	3206	3.19	994	4663	1.98	1.85
2016	435	3680	2.82	431	5529	0.99	2.54
2015	349	4524	3.24	304	3713	0.87	2.13
2014	284	4131	2.91	140	2914	0.49	2.05
2013	268	5167	3.21	118	2893	0.44	2.16
2012	202	4642	3.28	66	1511	0.33	1.50
2011	173	4706	3.40	64	1150	0.37	1.33
2010	146	5474	4.17	51	881	0.35	1.21
2009	114	3550	3.11	55	729	0.48	1.28
2008	88	3671	3.79	36	478	0.41	1.09
2007	68	2480	3.04	24	388	0.35	1.14
2006	58	2324	3.08	18	238	0.31	0.82
2005	45	1885	2.99	14	219	0.31	0.97
2004	26	1582	4.06	6	134	0.23	1.03
2003	39	3115	4.99	22	399	0.56	2.05
2002	17	3208	11.10	32	297	1.88	3.49
2001	15	964	3.57	2	75	0.13	1.00
2000	18	2040	5.96	11	192	0.61	2.13
1999	13	1043	4.01	5	51	0.38	0.78
1998	12	548	2.17	4	31	0.33	0.52
1997	9	420	2.12	5	28	0.56	0.62
1996	2	52	1.13	0	4	0.00	0.40
1995	2	297	6.19	5	28	2.50	2.80
1994	4	172	1.72	0	9	0.00	0.45
1993	0	0	0	0	0	0.00	0.00
1992	3	105	1.30	2	6	0.67	0.40
1991	1	2	0.07	0	2	0.00	0.40

^a^Mean citation rate per year=total citations/(total citations×[2018−that year]).

^b^Total usage: total downloads.

^c^Mean use rate for the last 6 months=total usage in the last 6 months/total number of papers.

^d^Mean use rate for the last 5 years=total usage in the last 5 years/(total number of papers×5).

**Table 3:** Number of papers by countries as study settings (N=749).

Rank	Country settings	Frequency, n (%)
1	United States	348 (46.5)
2	Ireland	47 (6.3)
3	Taiwan	44 (5.9)
4	Japan	41 (5.5)
5	United Kingdom	37 (4.9)
6	India	33 (4.4)
7	China	25 (3.3)
8	Australia	22 (2.9)
9	Italy	14 (1.9)
10	Mali	12 (1.6)
11	Sweden	11 (1.5)
12	Wallis and Futuna	10 (1.3)
13	Germany	9 (1.2)
14	Netherlands	9 (1.2)
15	Poland	9 (1.2)
16	France	7 (0.9)
17	Spain	7 (0.9)
18	Denmark	6 (0.8)
19	Hong Kong	6 (0.8)
20	Canada	5 (0.7)
21	Finland	5 (0.7)
22	Iran	5 (0.7)
23	Singapore	4 (0.5)
24	Belgium	3 (0.4)
25	Brazil	3 (0.4)
26	Egypt	3 (0.4)
27	Israel	3 (0.4)
28	Malaysia	3 (0.4)
29	Turkey	3 (0.4)
30	New Zealand	2 (0.3)
31	Norway	2 (0.3)
32	Antarctica	1 (0.1)
33	Austria	1 (0.1)
34	Georgia	1 (0.1)
35	Greece	1 (0.1)
36	Iceland	1 (0.1)
37	Indonesia	1 (0.1)
38	Jersey	1 (0.1)
39	Jordan	1 (0.1)
40	Pakistan	1 (0.1)
41	Saint Pierre	1 (0.1)
42	Saudi Arabia	1 (0.1)

Figure 1 presents the global network among 53 countries having at least five co-authorships with other countries. The range of nodes represents the contribution of each country to the total number of publications, and the thickness of lines indicates the proportion of the volume of collaborations. These countries were classified into 8 clusters depending on their level of international collaborations.

**Figure 2:**
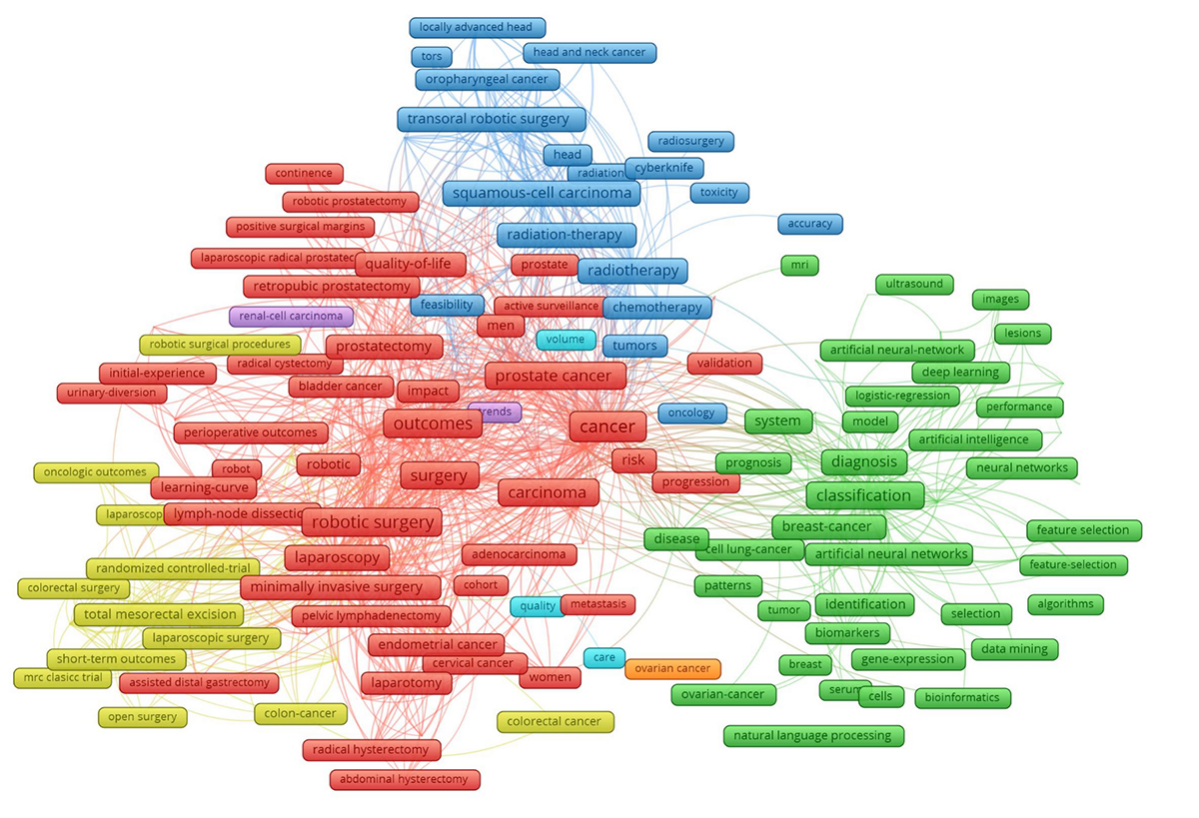
Co-occurrence of the most frequent author’s keywords.

Analyses of keywords and abstract contents provide us with a better understanding of the scopes of studies and development of the research landscapes. Figure 2 describes the co-occurrence of keywords with the most frequent groups of terms. There were 8 major clusters emerging from 180 most frequent keywords with a co-occurrence of 30 times and higher. Some major clusters included the following: Cluster 1 (red) refers to surgery and treatment outcomes; Cluster 2 (green) focuses on the applications of AI techniques in some specific cancers; Cluster 3 (yellow) describes the therapies for colorectal cancers; and Cluster 4 (blue) illustrates applications of chemotherapy and radiotherapy. The colors of the nodes indicate principal components of the data structure; the node size was scaled to the keyword occurrences; and the thickness of the lines is based on the strength of the association between 2 keywords.

As for the content analysis of abstracts, the top 50 emerging research domains are listed in Table 4. AI techniques have been applied to various aspects of cancer research, including therapies (radiotherapy, chemotherapy, and surgery), capacities (prediction, screening, and treatment), and factors associated with outcomes (physical, social, and economic).

**Figure 3:**
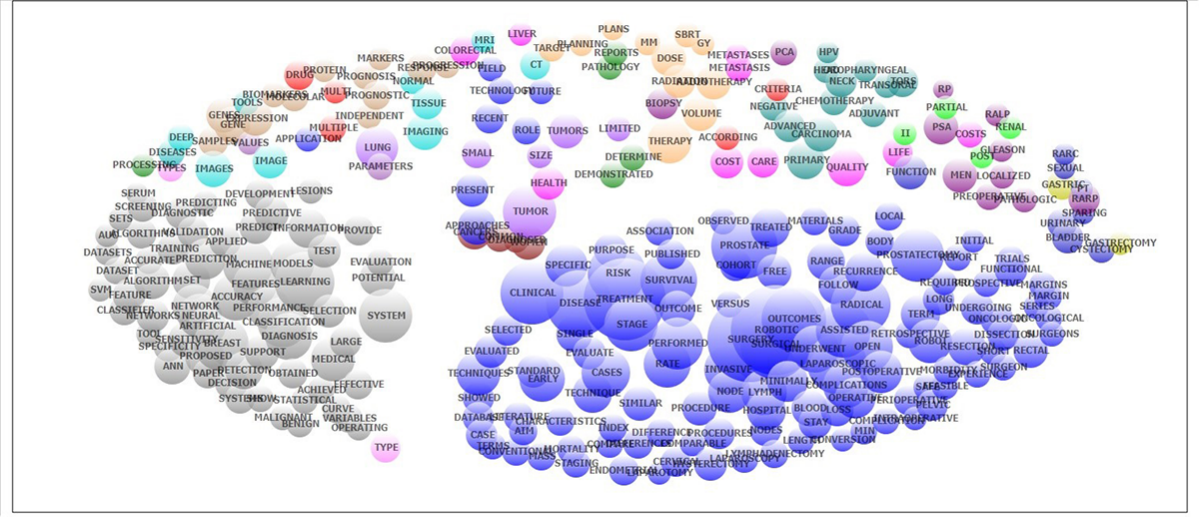
Co-occurrence of most frequent topics emerged from exploratory factor analysis of abstracts contents.

Figure 3 illustrates the classification of the co-occurrence of research domains into principal components. Primarily, we have the following major landscapes: (1) robotic surgery (blue), (2) AI techniques for detection and prediction (gray), (3) chemotherapy (jade), and (4) radiotherapy (yellow).

**Figure 4:**
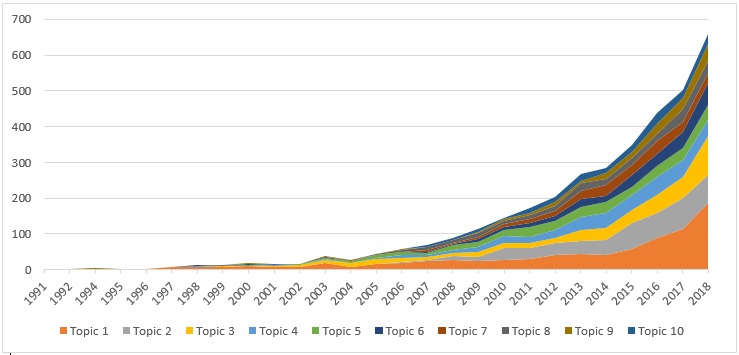
Changes in the applications of artificial intelligence to cancer research during 1991-2018.

In Table 5, we present the research topics that were constructed using LDA. The labels of the topics were manually annotated by scrutinizing the most frequent words and titles for each topic. Topics with the highest volume of publications included (1) machine learning, (2) comparative effectiveness evaluation of AI-assisted medical therapies, and (3) AI-based prediction. Noticeably, this classification has revealed topics examining the incremental effectiveness of AI applications (Topic 2) and, more interestingly, the quality of life outcomes and functioning of patients receiving these innovations. The changes in research productivity over time are illustrated in Figure 4, which shows the rapid growth of Topics 1, 2, 3, and 4, especially in recent years.

**Table 4:** Top 50 research domains that emerged from the exploratory factor analysis of the content of all abstracts.

Number	Name	Keywords	Eigen value	Cases, n (%)
1	Classification; feature selection	Classification; feature; proposed; features; selection; breast; performance; algorithm; diagnosis; classifier; paper; accuracy; machine	4.71	6104 (58.09)
2	Disease-free survival	Survival; free; recurrence; follow; disease; local	2.94	3413 (52.69)
3	Medical; processing	Medical; processing; information; system; systems	1.85	2218 (42.31)
4	Blood loss; hospital stay	Loss; blood; stay; operative; complications; length; hospital; min; postoperative; conversion; perioperative; complication; safe; intraoperative; feasible	17.16	5050 (41.13)
5	Prostate; assisted radical	Prostatectomy; prostate; radical; RARP^a^; localized; men; assisted; radical prostatectomy; robot; Gleason; RALP^b^; PSA^c^	3.97	4050 (41.04)
6	Gy; radiation dose	Gy; dose; SBRT^d^; radiation; radiotherapy; therapy; local; body; treated	5.18	2495 (37.16)
7	Predict; prediction	Predict; prediction; predictive; models; predicting; prognostic; variables; validation	2.34	2562 (36.71)
8	Machine learning	Learning; machine; accuracy	2.27	2398 (36.68)
9	Cohort; risk	Cohort; risk; outcome; retrospective	1.44	1759 (36.54)
10	PSA; Gleason	PSA; Gleason; specific; biopsy; serum; prostate	1.38	1970 (34.04)
11	Early stage	Early; cervical; stage; hysterectomy	1.48	1700 (33.47)
12	Evaluate	Evaluate; evaluated; according	1.37	1185 (28.16)
13	Training set	Training; set; test; sets; validation	1.60	1573 (27.74)
14	Adjuvant chemotherapy	Chemotherapy; adjuvant; therapy; advanced	1.51	1323 (27.34)
15	Tumor	Tumor; tumors; size	1.46	1253 (27.12)
16	Morbidity and mortality	Mortality; morbidity; rate	1.36	1194 (26.69)
17	Staging for endometrial; hysterectomy	Endometrial; hysterectomy; laparotomy; staging; lymphadenectomy; pelvic; cervical; laparoscopy; women	3.09	1759 (25.26)
18	Sensitivity and specificity	Specificity; sensitivity; serum; detection; diagnostic	2.17	1405 (23.74)
19	Plans; planning	Plans; planning; target; mm; volume; dose; average	1.53	1267 (23.57)
20	Cystectomy; bladder	Cystectomy; bladder; RARC^e^; urinary; radical	2.83	1235 (22.53)
21	Case	Cases; case	1.26	913 (22.50)
22	Artificial neural	Neural; artificial; network; ANN^f^; networks	3.32	2060 (22.17)
23	Image	Images; image; imaging; deep; CT^g^; MRI^h^	2.59	1363 (21.77)
24	Quality of life	Life; quality; health; sexual	2.09	1148 (21.55)
25	Lymph node	Lymph; node; dissection; nodes; pelvic; lymphadenectomy	2.47	1909 (21.24)
26	Safe and feasible	Safe; feasible; procedure	1.25	1020 (20.39)
27	Decision support	Support; SVM^i^; decision; classifier	1.45	1062 (19.86)
28	Rectal resection	Rectal; colorectal; resection; conversion	1.57	926 (19.16)
29	Oncological and functional; sexual function	Functional; function; sexual; oncological	1.31	903 (19.10)
30	Gene expression	Gene; expression; genes; molecular; protein; samples; mutations	3.24	1369 (18.90)
31	Purpose	Purpose; materials	1.51	815 (18.71)
32	Pathology; reports	Pathology; reports; processing; report	1.41	824 (18.54)
33	Women diagnosed	Diagnosed; screening; women	1.29	728 (17.64)
34	Transoral; TORS^j^	Transoral; tors; oropharyngeal; neck; head; HPV^k^; carcinoma	3.48	1183 (16.48)
35	Margin; PT^l^	Margin; PT; margins; pathologic; Gleason; RALP	1.80	884 (16.15)
36	Cost	Cost; costs; care	1.93	712 (16.06)
37	Surgeon experience	Experience; surgeons; surgeon	1.70	690 (15.22)
38	Small cell lung	Lung; small	1.29	628 (14.91)
39	Body mass	Mass; index; body	1.89	823 (14.91)
40	Multi drug	Multiple; multi; drug	1.56	588 (14.68)
41	Operating curve	Curve; operating; AUC^m^	1.68	649 (12.52)
42	Benign and malignant	Malignant; benign; lesions	1.59	579 (11.14)
43	Normal tissue	Tissue; normal	1.40	460 (11.11)
44	Imaging (MRI)	MRI; imaging	1.39	435 (10.63)
45	Metastases	Metastases; metastasis; liver	1.39	416 (9.51)
46	Renal	Renal; partial; sparing	1.74	376 (8.27)
47	HPV-negative	Negative; HPV	1.33	312 (8.07)
48	Trials	Trials	1.34	239 (6.72)
49	Biomarkers	Biomarkers	1.25	171 (4.81)
50	Gastrectomy for gastric	Gastric; gastrectomy	1.77	205 (3.63)

^a^RARP: robotic-assisted radical prostatectomy.

^b^RALP: robot assisted laparoscopic prostatectomy.

^c^PSA: prostate specific antigen.

^d^SBRT: stereotactic body radiation therapy.

^e^RARC: remittance advice remark code.

^f^ANN: artificial neural network.

^g^CT: computed tomography.

^h^MRI: magnetic resonance imaging.

^i^SVM: support vector machine.

^j^TORS: transoral robotic surgery.

^k^HPV: human papilloma virus.

^l^PT: prothrombin time.

^m^AUC: area under the curve.

**Table 5:** Ten research topics classified by Latent Dirichlet Allocation.

Topics	Research areas	Frequency (N=3555), n (%)
Topic 1	Machine learning	824 (23.18)
Topic 2	Comparative effectiveness evaluation of AI^a^-assisted medical therapies	513 (14.43)
Topic 3	AI-based prediction	456 (12.83)
Topic 4	Multidisciplinary care, precision, and personalized medicine	371 (10.44)
Topic 5	Quality of life outcomes, physical and mental health, and functioning	312 (8.78)
Topic 6	Enhanced radiotherapy	270 (7.59)
Topic 7	Robotic surgery	229 (6.44)
Topic 8	AI-assisted imaging and signals	215 (6.05)
Topic 9	Data mining and natural language processing	183 (5.15)
Topic 10	AI and robotic-assisted cancer diagnosis and therapies	182 (5.12)

^a^AI: artificial intelligence.

**Figure 5:**
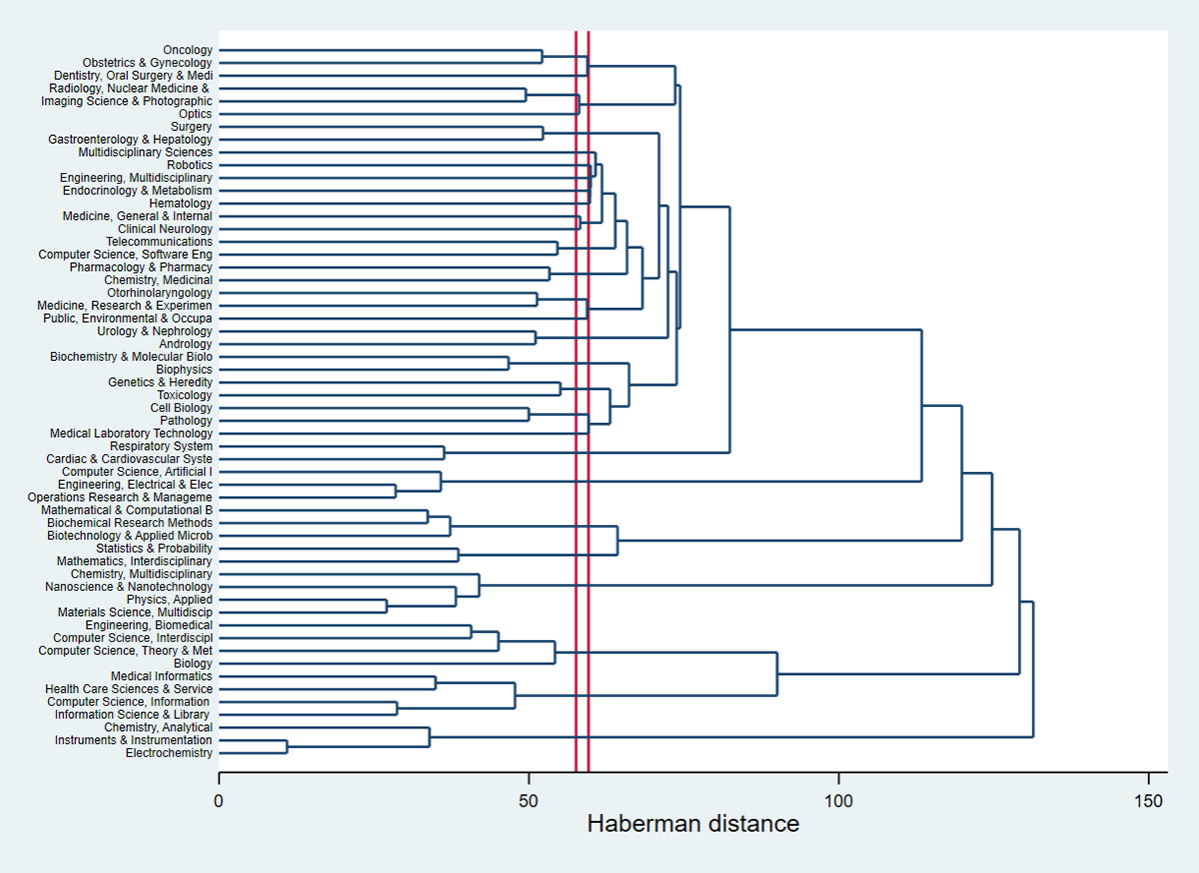
Dendogram of coincidence of research areas using the Web of Science classifications.

Figure 5 presents the hierarchical clustering of research disciplines used in AI and cancer research. The horizontal axis of the dendrogram represents the distance or dissimilarity between clusters. The vertical axis represents the research disciplines. It shows that AI applications in cancer care are rooted in the following disciplines: robotics, multidisciplinary engineering, and multidisciplinary sciences. Imaging science and photography was very close to oncology, obstetrics and gynecology, dentistry, radiology, and optics. Those biomedical and clinical aspects account for the major areas of AI application; meanwhile, health service–focused areas, for example, operations and management, are rather distant.

## Discussion

### Principal Findings

By systematically synthesizing and analyzing the bibliography of AI applications in cancer studies, we have characterized the development of its research landscape over the period from 1991 to 2018. The findings illustrate the rapidly growing research productivity and expansion of multidisciplinary approaches, largely driven by machine learning, artificial neural networks, and AI in various clinical practices. Our analysis highlights the most frequent areas of research and the paucity of research in other areas. The research topics and landscapes constructed show that the development of AI in cancer care is focused on improving prediction in cancer screening and AI-assisted therapeutics and corresponding areas of precision and personalized medicine. Our findings show the rapid growth in these areas over the past decade. Although cancer outcomes of interest covering clinical and physical functioning and mental and quality of life measures are on the rise, our analysis indicates the relative paucity of research focusing on cancer outcomes and survivorship. This is of special relevance, considering the continuously growing cancer survivor population [22].

### Comparison With Past Work

This study supplements the previous global mapping on AI in medicine by analyzing the content and characteristics of studies of specific applications of AI in cancer research and clinical practice [2]. Compared with previous reviews, this study is more comprehensive in describing the research trends by applying content analysis and topic modeling [4-10]. Therefore, the findings are helpful to inform the design and priority of the settings of future studies. Classifying information sources and content in corresponding topics to identify priorities for interventions has been widely applied in many studies. For example, previous authors have analyzed newspaper and social media content to understand topics of interest related to breast cancer and secondhand smoking [23-28]. However, none of the previous studies have analyzed the scientific bibliography to determine the development of research landscapes in AI applied in cancer care. Li et al proposed a text-mining framework using LDA to construct topics that were helpful for supporting systematic reviews [29]. In this study, we applied this approach to classify topics that a paper belongs to. Moreover, we further analyzed the frequency of concurrence of terms and their associated clusters using factor analysis. These clusters of terms enrich the understanding of scopes of each topic, especially for diseases involving the development of multidisciplinary research.

The findings from this study help inform the future development of AI applications in cancer research and clinical practices of cancer control and management. First, the difference in citation rates between very recent articles and older articles demonstrates the speed of knowledge accumulation in this area. Understanding the scope of research landscapes helps inform the selection of variables and topics to develop an application or conduct a study. Moreover, the previous bibliometric analysis could only distinguish and determine trends in the applications of AI techniques in cancer care, whereas this study showed that research trends have also expanded to encompass the comparative effectiveness of these innovations compared with traditional practices [2]. In addition, research landscapes have expanded beyond clinics to evaluate the functioning and performance of the patients being treated, in addition to their mental well-being and quality of life. To support this research topic, there should be more exploration of different study settings and incorporation of individual characteristics to improve the validity of AI techniques. One important question is how to integrate and scale-up AI-based applications in cancer care into clinical practice and community prevention. Currently, little is known on the adaptation and integration of AI applications into health systems and communities; future implementation research should be conducted.

### Limitations

One of the shortcomings of this study is that we used only WOS databases. Although the WOS covers the greatest proportion of the literature in the field of AI research, it might not be fully representative of all databases. Another limitation is that only documents in English were selected for this study. Finally, the content analysis included only abstracts instead of full texts. Nonetheless, this topic modeling serves to expand, improve, and supplement previous systematic reviews in this field.

### Conclusions

In conclusion, AI applications have been rapidly growing in cancer clinical practices, including prediction, diagnosis, enhanced therapeutics, and optimal selection. As interest in AI in medicine continues to grow, it will be increasingly critical to better understand the incremental effectiveness of these innovations and their validities in supporting the performance and quality of life of individuals after getting treated.

## Abbreviations

AI: artificial intelligence
ANN: artificial neural network
AUC: area under the curve
CT: computed tomography
HPV: human papilloma virus
LDA: Latent Dirichlet Allocation
MRI: magnetic resonance imaging
PSA: prostate specific antigen
PT: prothrombin time
RALP: robot assisted laparoscopic prostatectomy
RARC: remittance advice remark code
RARP: robotic-assisted radical prostatectomy
SBRT: stereotactic body radiation therapy
SVM: support vector machine
TORS: transoral robotic surgery
WOS: Web of Science
